# *Sarcodia suieae* acetyl-xylogalactan regulate RAW 264.7 macrophage NF-kappa B activation and IL-1 beta cytokine production in macrophage polarization

**DOI:** 10.1038/s41598-019-56246-9

**Published:** 2019-12-23

**Authors:** Tsung-Meng Wu, Fan-Hua Nan, Kuan-Chu Chen, Yu-Sheng Wu

**Affiliations:** 1 Department of Aquaculture, National Pingtung University of Science and Technology, Pingtung, Taiwan; 2 Department of Aquaculture, National Taiwan Ocean University, Keelung, Taiwan

**Keywords:** Polysaccharides, Biomaterials - cells

## Abstract

In this study, the effects of acetyl-xylogalactan extracted from *Sarcodia suieae* on RAW 264.7 macrophage polarisation were evaluated. This extracted acetyl-xylogalactan had a monosaccharide composition of 91% galactose and 9% xylose, with polysaccharide and acetyl contents of 80.6% and 19.3%, respectively. MALDI–TOF mass spectrometry and NMR spectroscopy revealed the molecular weight of the acetyl-xylogalactan to be 88.5 kDa. After acetyl-xylogalactan treatment, RAW 264.7 macrophage polarisation was noted, along with enhanced phagocytic ability. Furthermore, the Cell Counting Kit-8 (CCK-8) assay was performed and the results demonstrated non-significant alteration in lactate dehydrogenase levels in the treated cells. Next, interleukin (IL) 1β, TNF, and Malt-1 expression in RAW 264.7 macrophages treated with the *S. suieae* acetyl-xylogalactan was investigated through real-time quantitative polymerase chain reaction, and the results demonstrated that *S. suieae* acetyl-xylogalactan induced IL-1β and Malt-1 expression. RNA sequencing analysis results indicated the *S. suieae* acetyl-xylogalactan positively regulated cytokine production and secretion, protein secretion, and response to IL-1 activation, based on the observed GO terms. The predicted target genes in the GO enrichment analysis were found to upregulate NF-κB signalling and M0 to M1 macrophage conversion through the observed cytokine production. Thus, acetyl-xylogalactan can positively regulate RAW 264.7 macrophage polarisation.

## Introduction

Innate immune cells, such as macrophages, dendritic cells, and other nonimmune cells, are involved in the response to invading pathogens^1^. The administration of algal polysaccharides can enhance tumour-suppressing and antiallergic abilities by modulating the host immune system^2,3^. Moreover, algal polysaccharides significantly enhance wound-healing ability^4^.

Algae have been used for thousands of years^5^. Marine macroalgae, such as red and green algae, contain high levels of protein, polysaccharides, long-chain fatty acids, and other biocompounds^6^. Marine algal polysaccharides can aid energy storage and basal structure maintenance^7^.

Many studies have reported the bioactivity of polysaccharides from algae, such as *Arthrospira*, *Dunaliella*, *Haematococcus*, *Scenedesmus*, and *Sarcodia*^8–11^. These polysaccharides have demonstrated positive in antitumour, cytokine-regulatory, and antiinflammatory bioactivity. Polysaccharides typically bind to dectin-1 and then activate the immune cell, such as macrophages, neutrophils, and dendritic cells^12^. Dectin-1, a C-type lectin family receptor, is expressed on cell membranes^13^. Dectin-1 binding to polysaccharides promotes phagocytosis, induces ROS production, enhances neutrophil degranulation, and induces immune cells to regulate cytokine production in macrophages^14^. The induced cytokines act as signalling moleculus that initiate cellular alteration.

Moreover, polysaccharides bind to the complement receptor type 3 (CR3)^15^. This receptor, expressed on immune cells, identifies polysaccharides^16^. When recognised, CR3 induces signalling to activate transcription factors. The major function of these transcription factors is inflammatory cytokine regulation.

Algal immunoregulatory substances include polysaccharides and proteins. However, to our knowledge, the mechanisms of most bioactive immunoregulatory substances involved in macrophage polarisation are somewhat unknown. This study investigated whether acetyl-xylogalactan from *Sarcodia suieae* induces macrophage polarisation. First, the monosaccharide and polysaccharide contents, monosaccharide composition, acetyl content, and molecular weight of this acetyl-xylogalactan were evaluated through MALDI–TOF mass spectrometry and NMR spectroscopy. Next, its effect on RAW 264.7 macrophage morphology alteration, phagocytic activity change, and interleukin (IL) 6 and IL-17A production was examined. Moreover, the treated RAW 264.7 macrophages were analysed to predict signal transduction through next-generation sequencing (NGS) and the related gene expression was examined using real-time qPCR.

## Materials and Methods

### *S. suieae* acetyl-xylogalactan extraction and analysis

**Figure 1:**
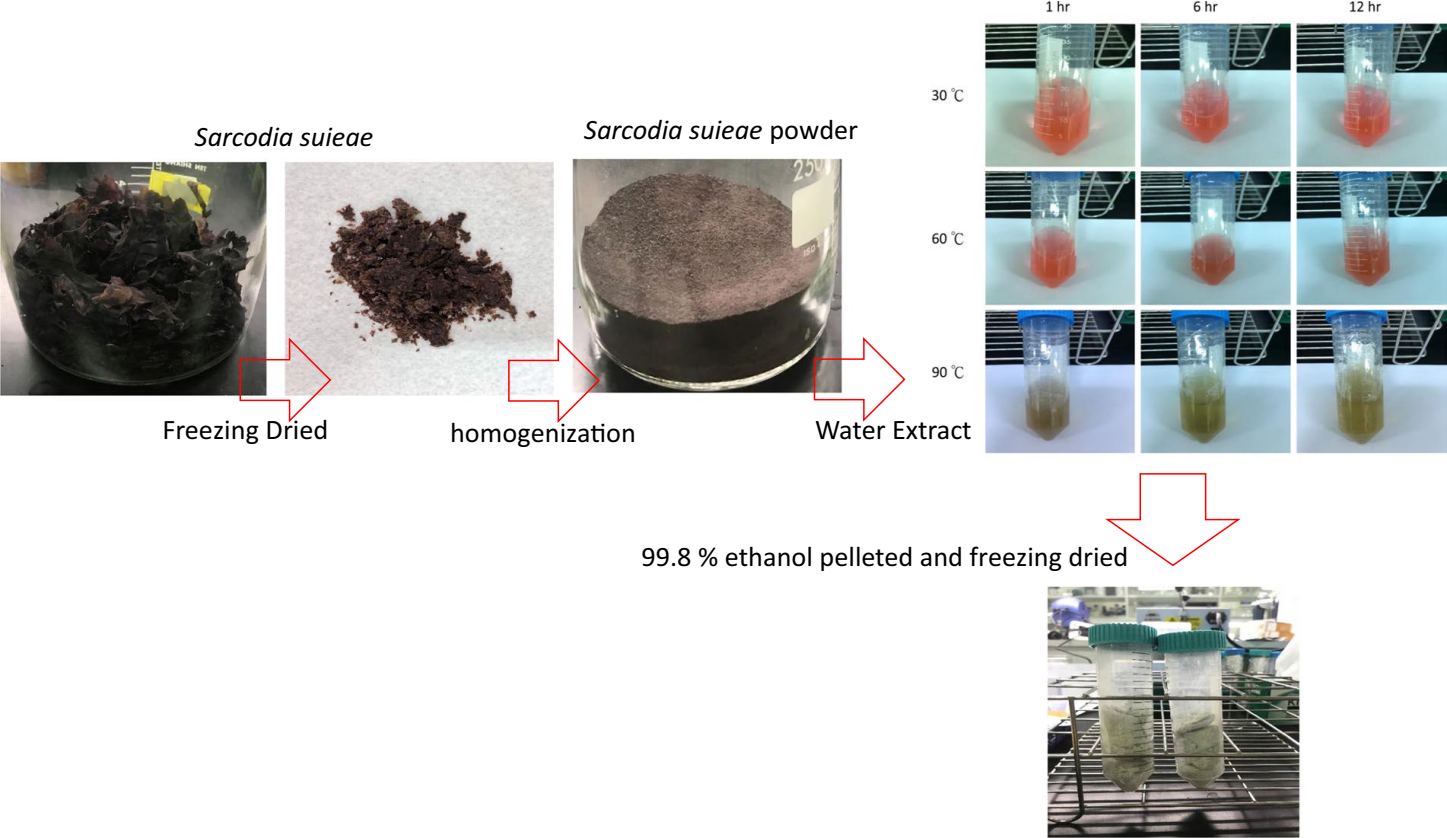
Process of acetyl-xylogalactan extraction from *S. suieae*.

*S. suieae acetyl-xylogalactan* was extracted from laboratory. *S. suieae* was collected from a commercial sea algae culture pond in Southern Taiwan. The collected *S. suieae* were freeze-dried and homogenised to a powder. This powder was soaked in water of various temperatures (30 °C, 60 °C, and 90 °C) for various periods (1, 6, and 12 h) at a powder-to-double distilled water ratio of 1:40, so as to extract the water-soluble materials. Next, the sample was centrifuged at 4000 rpm for 30 min to collect the supernatant. The extracted polysaccharide in the supernatant was pelleted using 99.8% water-free ethanol (Sigma) (supernatant:water-free ethanol = 1:3) for 2 h. The polysaccharide extraction procedure is presented in Fig. 1.

The monosaccharide and polysaccharide contents and monosaccharide components were analysed through MALDI–TOF mass spectrometry and NMR spectrometry^17^. The acetyl content and molecular weight were evaluated through NMR spectrometry^18,19^ by Sugarlighter, Taiwan.

### RAW 264.7 macrophage culture

RAW 264.7 macrophages [Bioresource Collection and Research Center (BCRC) No. 60001] were purchased from BCRC, Food Industry Research and Development Institute, Hsinchu, Taiwan. After the cells were received by our laboratory, they were maintained in 90% Dulbecco’s modified Eagle’s medium with 10% foetal bovine serum, incubated at 37 °C and 5% CO_2_.

### Toxicity effect of *S. suieae* acetyl-xylogalactan on RAW 264.7 macrophages

To examine the toxicity of *S. suieae* acetyl-xylogalactan to RAW 264.7 macrophages, the experiment groups were classified as control receiving no treatment and treatment groups treated with 10, 20, and 30 μg/mL *S. suieae* acetyl-xylogalactan.

In brief, RAW 264.7 macrophages (1 × 10^6^ cells) were treated with or without *S. suieae* acetyl-xylogalactan for 12 and 24 h. At the end of the treatment, the cells were observed using the Cell Counting Kit-8 (CCK-8; B34302, Bimake) at an OD of 450 nm.

### Effect of *S. suieae* acetyl-xylogalactan on RAW 264.7 macrophages’ phagocytic activity

The phagocytic activity of RAW 264.7 macrophages was determined using pHrodo Green BioParticles Conjugate for Phagocytosis (P35366, Thermo Scientific). In brief, 1 × 10^6^ cells were cultured in the medium at 37 °C and 5% CO_2_ for 24 h. After incubation, 10, 20, and 30 μg/mL *S. suieae* acetyl-xylogalactan was added to the cells and then incubated for 24 h. Next, the culture medium was removed and bioparticles were added, followed by incubation for 2 h. At the end of the culture, the phagocytic activity was detected at Ex/Em of 509/533.

### IL-6 and IL-17A production in RAW 264.7 macrophages

IL-6 and IL-17A production in the RAW 264.7 macrophages treated with or without *S. suieae* acetyl-xylogalactan was analysed. In brief, 1 × 10^6^ RAW 264.7 macrophages were cultured in a 96-well plate with or without 10, 20, or 30 μg/mL *S. suieae* acetyl-xylogalactan at for 24 h. The culture medium was then analysed using the ELISA IL-6 and IL-17A assay kit (QIAGEM, SEM03015A and SEM03023A) at an OD of 450 nm.

### RNA sequencing (transcriptome)

RAW 264.7 macrophages (1 × 10^6^ cells) were treated with or without 10, 20, or 30 μg/mL *S. suieae* acetyl-xylogalactan for 24 h. Thereafter, RNA was isolated from the cells using Azol RNA Isolation Reagent (Arrowtech). RNA concentrations were determined using Nanodrop, and then, 1 µg of RNA was sent to Biotools Co., Ltd., Taiwan for the RNA sequencing (RNA-seq; transcriptome) analysis, where NovaSeq. 6000 Sequencing System (Illumina) is used. The reference genome mapped using HISAT2. Only filtered reads could be used to analyse the mapping status of RNA-seq data to the reference genome.

### Real-time reverse transcription qPCR for gene expression

Total RNA was extracted using Azol RNA Isolation Reagent (Arrowtech) and quantified through spectrophotometry at 260 nm. Real-time qPCR analysis of the macrophage IL-1β, TNF, and Malt-1 was performed by Biotools Co., Ltd.. GAPDH was used as the reference in the comparative CT to determine the relative alteration. Fluorescence was analysed using the auto CT method to determine the threshold of each gene, and the 2^−ΔΔCT^ method was used to calculate CT values by using StepOne (version 2.3). Data are presented as fold changes in the mRNA level normalised to the reference gene GAPDH.

The following oligonucleotide sequences were used for creating qPCR primers:

**Table:** 

Gene	Forward primer	Reverse primer
IL-1 b	TGGACCTTCCAGGATGAGGACA	GTTCATCTCGGAGCCTGTAGTG
TNF	GGTGCCTATGTCTCAGCCTCTT	GCCATAGAACTGATGAGAGGGAG
Malt-1	GAACTGAGCGACTTCCTACAGG	AACTGTCCAGCCAACACTGCCT
GAPDH	CATCACTGCCACCCAGAAGACTG	ATGCCAGTGAGCTTCCCGTTCAG

### Statistical analysis

Scheffé’s test and one-way ANOVA were used to analyse the statistical significance between the treatment and control groups. A p-value of <0.05 was considered statistically significant. The results are presented as the means ± SD (p < 0.05 and p < 0.001). For RNA-seq data, DEGseq was used to analyse significant differences between the treatment and control groups. A relative log expression of > 2 and a corrected p-value of < 0.005 were considered statistically significant.

## Results

### *S. suieae* acetyl-xylogalactan extraction and analysis

Acetyl-xylogalactan was extracted from *S. suieae* by using hot water and then extracted using ethanol (Fig. 1). In brief, the water-soluble materials were extracted from freeze-dried *S. suieae* powder by using hot water (60 °C) for 6 h, and then, acetyl-xylogalactan was obtained from the aqueous supernatant through 99.8% ethanol extraction.

The polysaccharide analysis results are presented in Table 1. The recovery rate for water extraction was 14%, followed by a polysaccharide recovery rate was 9% at 60 °C for 6 h. Although we performed polysaccharide extraction at 30 °C, 60 °C, and 90 °C, only extraction at 60 °C for 6 h afforded a desirable monosaccharide composition of 91% galactose and 9% xylose (Table 2). Furthermore, its polysaccharide and acetyl contents were nearly 80.6% and 19.3%, respectively, and its molecular weight was 88.5 kDa. Thus, this relative pure acetyl-xylogalactan was selected for the further investigation.

**Table 1:** Water extraction results for *S. suieae* exposed to various temperatures for 1, 6, and 12 h.

Extraction Condition		Dried Weight (g)	Water Extract Materials Recovery Rate (%)	Ethanol precipitation							
Temp.	Time			99.8% ethanol Recovery Rate (%)	Polysaccharide Content (%)	Component of Monosaccharide (%)					
						Glucose	Fructose	Sucrose	Galactose	Xylose	Others
30 °C	1 hour	1.000	13%	5%	50.6	2.4	—	—	83.0	14.6	—
	6 hour		16%	9%	71.1	—	—	—	82.4	11.0	6.6
	12 hour		17%	6%	78.8	—	—	—	64.8	35.2	—
60 °C	1 hour		18%	6%	67.0	—	—	—	89.1	7.8	3.1
	6 hour		14%	9%	80.6	—	—	—	91.0	9.0	—
	12 hour		21%	8%	75.2	—	—	—	90.7	7.8	1.5
90 °C	1 hour		18%	9%	52.3	72	25	<3	—	—	—
	6 hour		19%	9%	71.2	45	3	52	—	—	—
	12 hour		22%	12%	73.4	74	<3	51	—	—	—

**Table 2:** Ethanol extraction of polysaccharide. Polysaccharide collection was performed at 60 °C for 6 h. ‘-’: not detected.

Dried Weight (g)	Water Extract Materials Recovery Rate (%)	Ethanol precipitation					
		99.8% ethanol Recovery Rate (%)	Polysaccharide Content (%)	Component of Monosaccharide (%)		Acetyl Content(%)	Molecular Weight (kDa)
1.000	14	9	80.6	Galactose	91	19.3	88.5
				Xylose	9		

### Toxicity of *S. suieae* acetyl-xylogalactan to RAW 264.7 macrophages

**Figure 2:**
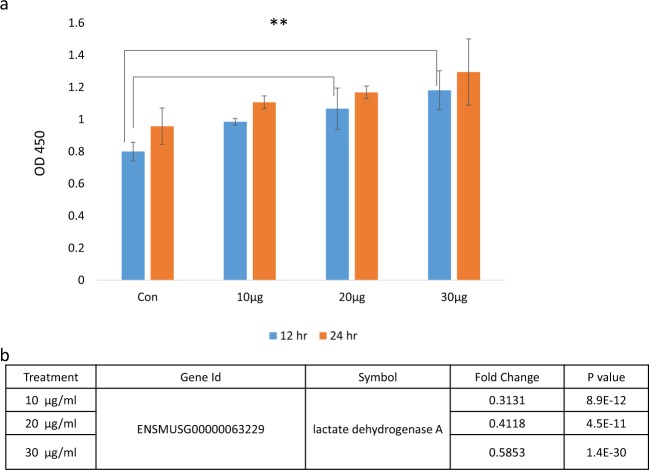
Toxicity assessment in RAW 264.7 macrophages treated with or without *S. suieae* acetyl-xylogalactan after 12 and 24 h incubation. *p < 0.05, **p < 0.01 (n = 5).

RAW 264.7 macrophages were treated with 10, 20, and 30 μg/mL acetyl-xylogalactan for 12 and 24 h and compared with untreated cells. The results indicated RAW 264.7 macrophages toxicity was not reduced in the experiment. After 12 h, the 20- and 30-μg/mL treatment groups demonstrated significant difference compared with the control group (p < 0.05; Fig. 2a), which diminished after 24 h (p > 0.05). Moreover, at 24 h, NGS detection of the fold changes in gene expression demonstrated significant induction of lactate dehydrogenase expression (p < 0.05). Thus, *S. suieae* acetyl-xylogalactan was not considered to be acutely toxic to RAW 264.7 macrophages.

Cellular toxicity was next observed using the CCK-8 assay, in which detection occurs when WST-8 [2-(2-methoxy-4-nitrophenyl)-3-(4-nitrophenyl)-5-(2,4-disulfophenyl)-2H-tetrazolium,monosodium salt] is reduced by dehydrogenases to form formazan (orange in colour). In this context, RNA-seq analysis demonstrated non-significant lactate dehydrogenase A expression after treatment concentration for 24 h (Fig. 2b).

### Effect of *S. suieae* acetyl-xylogalactan on RAW 264.7 macrophages’ phagocytic activity

**Figure 3:**
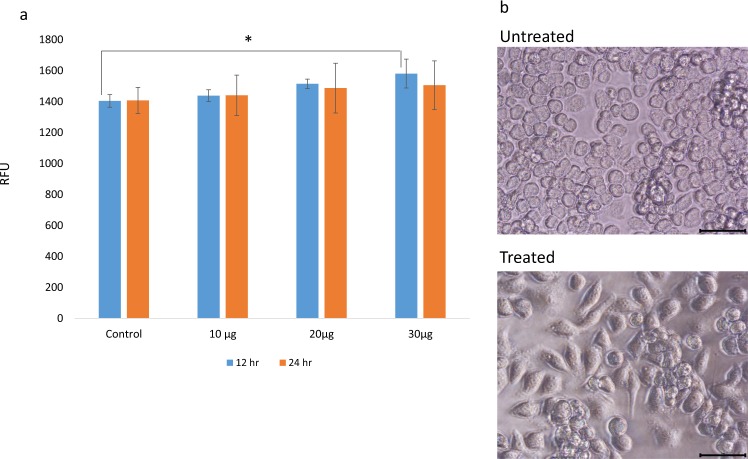
**(a)** Phagocytic ability of RAW 264.7 macrophages with or without *S. suieae* acetyl-xylogalactan treatment. *p < 0.05. **(b)** Microscopic observation of the phagocytosis by RAW 264.7 macrophages treated with or without *S. suieae* acetyl-xylogalactan. Black bar = 50 μm. (n = 5).

The effects of the acetyl-xylogalactan on RAW 264.7 macrophages’ phagocytic activity are presented in Fig. 3a. At 12 h, compared with control, 30 μg/mL acetyl-xylogalactan treatment significantly increased RAW 264.7 macrophages’ phagocytic ability (p < 0.05), but the effect of 10 and 20 μg/mL acetyl-xylogalactan was non-significant (p > 0.05). After 24 h, these changes in the phagocytic ability became non-significant in all groups (p > 0.05). Moreover, acetyl-xylogalactan treatment led to morphology alterations in RAW 264.7 macrophages: RAW 264.7 macrophages polarised to a fusiform shape was noted (Fig. 3b).

### IL-6 and IL-17A production of the RAW 264.7 macrophages

**Figure 4:**
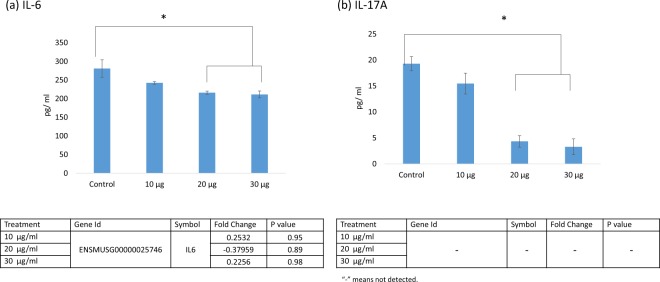
IL-6 and IL-17A production was observed from RAW 264.7 macrophages treated for 24 h. *p < 0.05, **p < 0.01 (n = 5).

Acetyl-xylogalactan regulated IL6 and IL 17A production in RAW 264.7 macrophages (Fig. 4). Compared with the control, IL-6 levels significantly decreased after treatment with 20 and 30 μg/mL acetyl-xylogalactan (p < 0.05), but this change was non-significant for 10 μg/mL acetyl-xylogalactan (p > 0.05; Fig. 4a). Moreover, compared with the other groups, IL-17A levels significantly decreased only after treatment with 30 μg/mL acetyl-xylogalactan (p < 0.05; Fig. 4b). At 24 h, RNA-seq for detection of the fold change in *IL6* expression also demonstrated nonsignificant differences for all groups (p > 0.05), but RNA-seq analysis for *IL17A* was not performed.

Thus, the extracted *S. suieae* acetyl-xylogalactan can regulate the production of macrophage cytokines, responsible for macrophage polarisation.

### RNA-seq (transcriptome) and real-time reverse transcription qPCR

RNA was extracted from RAW 264.7 macrophages and first mapped to the reference genome (Table 3). Multiple mapped reference genome counts were <10%, and the unique mapped counts to the reference genome were nearly 88%. In other words, the isolated RNA could be accurately mapped to the mouse reference genome.

**Table 3:** Mapping to Reference Genome.

Sample name	Con	10 μg/ml	20 μg/ml	30 μg/ml
Total reads	45082648	40039902	56093708	50170644
Total mapped	42730250 (94.78%)	37706492 (94.17%)	52873876 (94.26%)	47371721 (94.42%)
Multiple mapped	2749339 (6.10%)	2357681 (5.89%)	3239878 (5.78%)	2913806 (5.81%)
Uniquely mapped	39980911 (88.68%)	35348811 (88.28%)	49633998 (88.48%)	44457915 (88.61%)

Total reads: Total number of filtered reads; Total mapped: Total number of reads that could be mapped to the genome (this values is typically >70% when no contamination has occurred and the reference genome has been accurately selected); Multiple mapped: Total number of reads that could be mapped to multiple sites in the reference genome (should be <10% typically); Uniquely mapped: Total number of reads that could be uniquely mapped to the reference genome.

**Figure 5:**
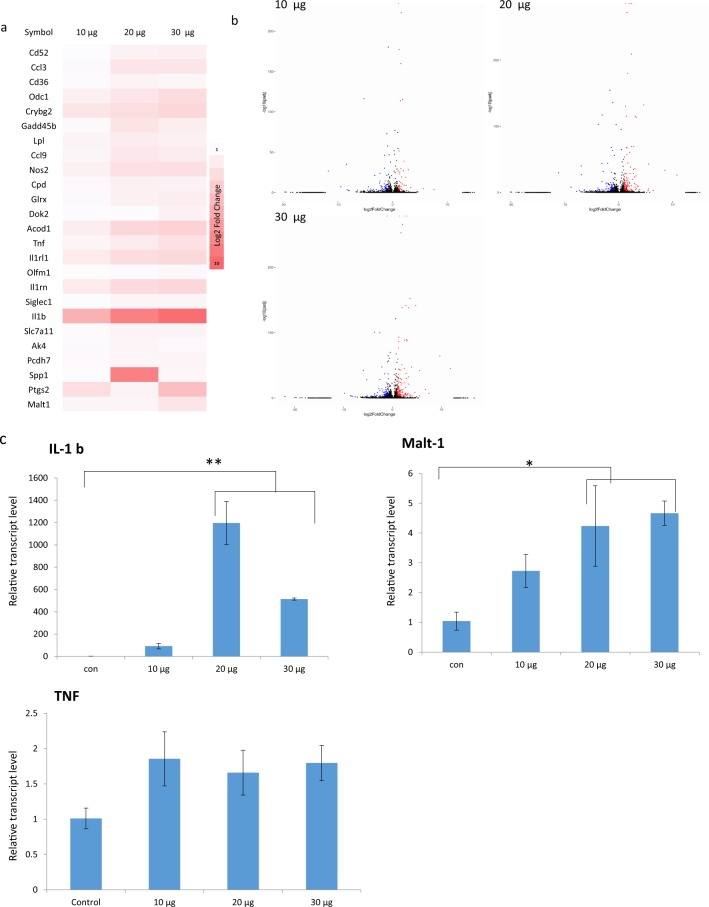
RNA-seq analysis of RAW 264.7 macrophages treated for 24 h. **(a)** Heatmap of the principal component analysis (PCA). **(b)** Volcano map of gene expression fold change and p-value significance change. **(c)** Real-time qPCR of *IL1B*, *TNF*, and *MALT1* expression (n = 3). C_10, C_20, and C_30 indicate 10, 20, and 30 μg/mL *S. suieae* acetyl-xylogalactan treatment, respectively.

RNA-seq analysis further revealed that *S. suieae* acetyl-xylogalactan treatment significantly altered the RNA gene expression. The log2 fold change in gene expression is presented in Fig. 5a. Acetyl-xylogalactan treatment increased RNA gene expression of *TNF*, *IL1B*, *MALT1*, and other genes in RAW 264.7 macrophages, as presented in the heatmap in Fig. 5a.

A volcano map (Fig. 5b) of the biological effects (log2 fold change) and their statistical significance (−log10 p-value) was used to compare the alteration in gene expression in the treatments with control. In the volcano map, the red spots represent the differentially expressed genes significantly upregulated by the treatment (p < 0.005, log2 fold change > 2). Real-time qPCR results for the IL-1β, TNF and Malt-1 terms are presented in Fig. 5c. M1 macrophage conversion was observed in RAW 264.7 macrophages treated with *S. suieae* acetyl-xylogalactan: 20 and 30 μg/mL acetyl-xylogalactan treatments significantly increased IL-1β expression nearly 400- and 600-fold, respectively (both p < 0.01). For Malt-1 expression, the relative transcript level increased approximately sixfold in the 20 and 30 μg/mL acetyl-xylogalactan treatment groups (p < 0.05). However, TNF expression did not significantly differ between the control and treatment groups (p > 0.05).

**Figure 6:**
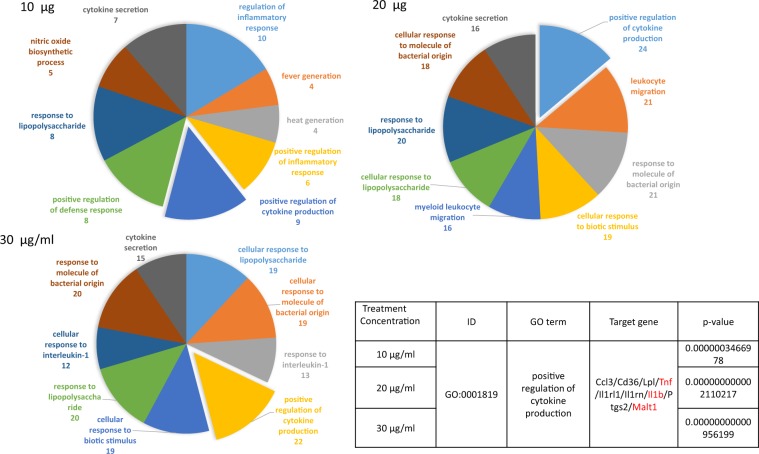
GO term prediction demonstrating *S. suieae* acetyl-xylogalactan facilitated the regulation of cytokine production. Predicted expressed genes included *CCL3*, *CD36*, *LPL*, *TNF*, *IL1RL1*, *IL1RN*, *IL1B*, *PTGS2*, and *MALT1*.

The predicted expressed genes *CCL3*, *CD36*, *LPL*, *TNF*, *IL1RL1*, *IL1RN*, *IL1B*, *PTGS2*, and *MALT1* had positive functions in the regulation of cytokine production, as presented the GO term analysis (Fig. 6).

**Figure 7:**
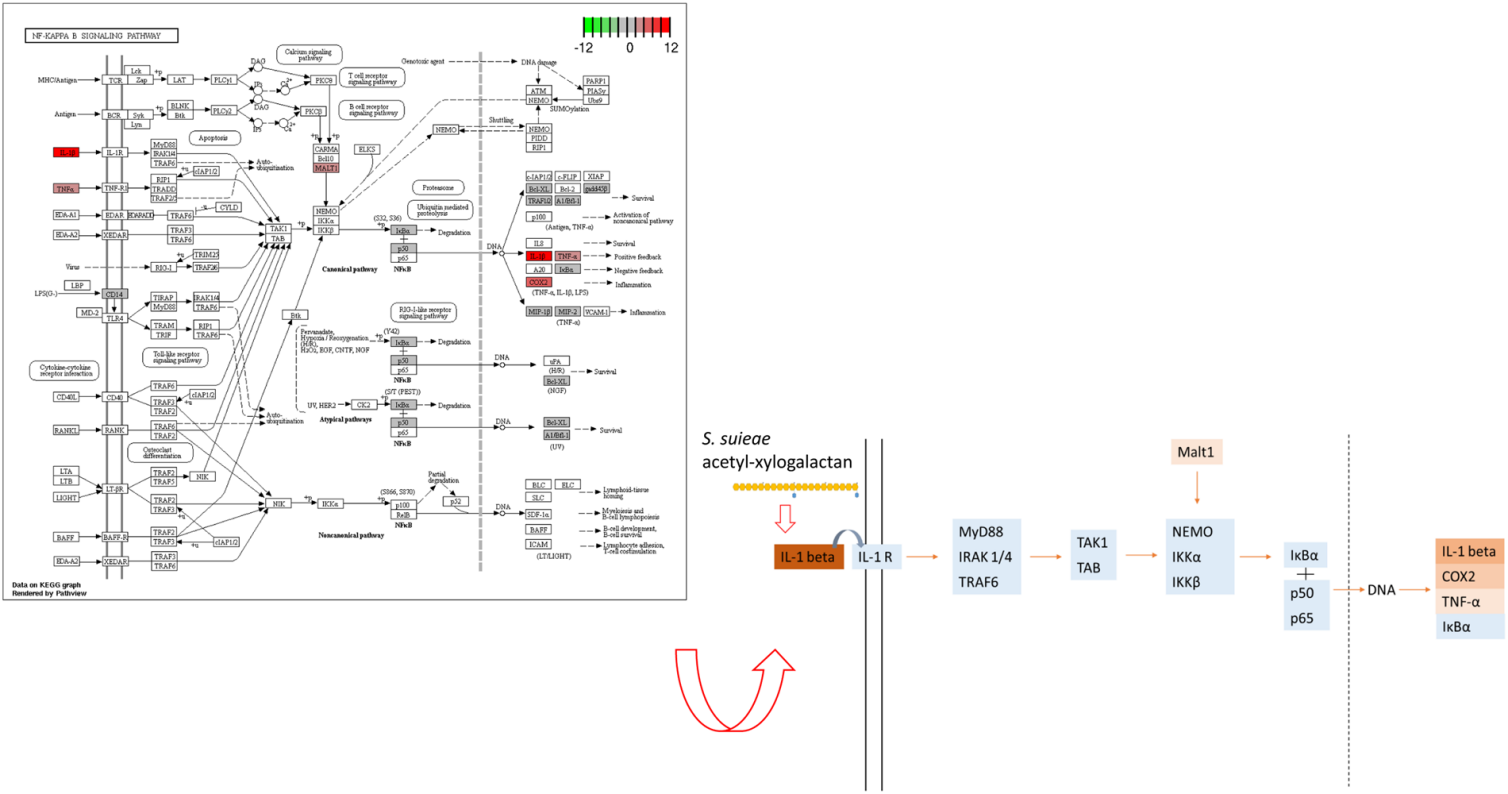
Predicted pathway illustrated that *IL1B* and *MALT1* were significantly upregulated in the NF-kappa B signalling pathway.

Taken together, these findings indicate that *S. suieae* acetyl-xylogalactan aided the M0 to M1 macrophage conversion. Pathway analyses revealed significantly upregulation of NF-kappa B signalling pathway components. Moreover, *S. suieae* acetyl-xylogalactan induced the expression of *IL1B*, *TNF*, and *MALT1*, which are involved in signalling transduction processes, based on KEGG observations presented in Fig. 7.

## Discussion

In this study, the regulatory effects of *S. suieae* acetyl-xylogalactan were investigated in RAW 264.7 macrophages. RAW 264.7 macrophages were treated with various concentrations of the *S. suieae* acetyl-xylogalactan and then the cellular response was analysed using microscopic observation and RNA-seq methods. The findings revealed that *S. suieae* acetyl-xylogalactan positively regulates cytokine production and activates the NF-kappa B signalling pathway.

Studies have linked the signalling mechanisms to inflammation regulation: NF-κB signalling regulates expression of cytokines (e.g., IL-1, IL-6, IL-8, and TNF) and chemokines and modulates adhesion molecules and cell-cycle regulatory molecules^20^. NF-κB, a transcription factor, has several functions in macrophages^21^, categorised into M1 and M2 macrophages: M1 macrophages release pro-inflammatory cytokines, such as IL-1, IL-6, IL-12, and TNF, which regulate inflammation response^22^^.^ On stimulation, transforming growth factor-β-activated kinase 1 (TAK1) is first activated to induce the downstream kinase multisubunit IκB kinase complex (IKK) response^23^. Activated IKK can phosphorylate and degrade the NF-κB inhibitor, IκBα, and cause NF-κB activation^24^. Activated NF-κB is responsible for the conversion of M0 macrophages to M1 macrophages and for cytokine production.

A *Lycium barbarum* polysaccharide could promote TNF-α and IL-1β production^25^. A signalling pathway analysis revealed that it enhanced p38-MAPK phosphorylation and reduced JNK and ERK1/2 MAPK phosphorylation^26^. Moreover, a study on the effects of a purified *Laminaria japonica* polysaccharide on the cytokine production in RAW 264.7 macrophages demonstrated that TNF and IL-1β increased with sample concentration^26^. Additionally, NF-κB p65 levels significantly increased after the *Laminaria japonica* polysaccharide treatment^27^. According to our findings in RAW 264.7 macrophages treated with *S. suieae* acetyl-xylogalactan for 24 h, real-time PCR results revealed increased TNF, IL-1β, and Malt-1 levels, ELISA demonstrated reduced IL-6 and IL-17 levels. Other studies have reported that IL-6 production is typically involved in the host defence observed in the infection or wounded tissues during the acute-phase response. In the current study, *S. suieae* acetyl-xylogalactan treatment did not cause toxicity to RAW 264.7 macrophages; hence, IL-6 and IL-17A must have not been produced. Thus, *S. suieae* acetyl-xylogalactan possibly increases the production of inflammation cytokines, such as TNF, IL-1β, and Malt-1, but inhibits that of acute proinflammatory cytokines, such as IL-6 and IL-17A.

Acetylated *Bletilla striata* polysaccharide modulates macrophage activation and wound healing^28^. Addition of methyl, acetyl, sulphate, and phosphate groups to polysaccharides increases the complexity of their primary structure and enhances their biological functions^29^. Compared with the nonacetyl polysaccharide, acetyl polysaccharides have antioxidant abilities and can inhibit the β-carotene–linoleic acid system; they also increase TNF-α expression by approximately 25%. The proposed underlying immunomodulatory mechanisms of these acetyl groups involve their interaction with the specific receptors and stimulation of the macrophage activation^30^. In this study, *S. suieae* acetyl-xylogalactan contained 19.3% acetyl groups and had a molecular weight of 88.5 kDa. Regarding the relationship between the *S. suieae* acetyl-xylogalactan and immunomodulation activation, the acetyl groups possibly facilitates the maintenance of the polysaccharide structure and their interaction with the cell-specific receptors to finally activate RAW 264.7 macrophages.

This study investigated the cellular functions of RAW 264.7 macrophages treated with *S. suieae* acetyl-xylogalactan. In the RNA-seq analysis, the *S. suieae* acetyl-xylogalactan was demonstrated to have positively regulated cytokine production and secretion, protein secretion, and response to the IL-1 activation, based on the observed GO terms. Of the predicted target genes in the GO enrichment analysis, *CCL3*, *CD36*, *LPL*, *TNF*, *IL1RL1*, *IL1RN*, *IL1Β*, *PTGS2*, and *MALT1*, all involved in the NF-κB signalling pathway, were upregulated. Taken together, *S. suieae* acetyl-xylogalactan induced the NF-κB signalling pathway in macrophages by the KEGG database, thus eliciting an immune response^31^.

In conclusion, polysaccharide from the animals, plants, microorganisms and macro-algae was known with a functional biological activities^32^, such as the anti-virus^33^, anti-tumor^34^, and anti-oxidation^35^ effects. Summarization of our findings, we observed that the extracted *S. suieae* acetyl-xylogalactan might directly induce TNF, IL-1, and Malt1 production but reduces IL-6 and IL-17A production, resulting in regulating inflammation response via the NF-κB pathway. Here, RAW 264.7 macrophages treated with *S. suieae* acetyl-xylogalactan had increased phagocytic ability. Thus, *S. suieae* acetyl-xylogalactan potentially modulates RAW 264.7 macrophage activation and polarisation to M1 macrophages.

## Supplementary information

Supplementary information 
