# Supplementary material for: Sarcodia suieae acetyl-xylogalactan regulate RAW 264.7 macrophage NF-kappa B activation and IL-1 beta cytokine production in macrophage polarization
Source: Sci Rep. 2019 Dec 23;9:19627. doi: 10.1038/s41598-019-56246-9 (PMC6927982; doi:10.1038/s41598-019-56246-9)
Supplement: Supplementary file 1 — Supplementary information [file 41598_2019_56246_MOESM1_ESM.docx]

**Supplement Data**

***Sarcodia suieae* acetyl-xylogalactan regulate RAW 264.7 macrophage NF-kappa B activation and IL-1 beta cytokine production in macrophage polarization**

Tsung-Meng Wu^1^, Fan-Hua Nan^2^, Kuan-Chu Chen^1^ and Yu-Sheng Wu^1,*^

1. Department of Aquaculture, National Pingtung University of Science and Technology, Pingtung, Taiwan

2. Department of Aquaculture, National Taiwan Ocean University, Keelung, Taiwan

***Correspondence Author:** Ph.D. Yu-Sheng Wu (Department of Aquaculture, National Pingtung University of Science and Technology, 1, Shuefu Road, Neipu, Pingtung 91201, Taiwan.)

**Phone:** +886-8-7703202#6207

**E-mail address:** wuys0313@mail.npust.edu.tw

**Competing interests:** The authors declare no competing interests.

**Key word:** *Sarcodia suieae*, Acetyl-xylogalactan, Macrophage, Polarization, NF-kappa B signaling

Supplement table 1.

RNA-seq analysis of the predicted expressed genes log 2 Fold changes. This analysis data was the *S. suieae* acetyl-xylogalactan 10 μg/ml treatment compared to the control in the gene fold change.

| Symbol | log2Fold  Change | Symbol | log2Fold  Change | Symbol | log2Fold  Change |
| --- | --- | --- | --- | --- | --- |
| Cd52 | 1.116219 | Il1b | 5.633935 | Layn | 1.015504 |
| Ccl3 | 1.184175 | Slc7a11 | 1.133778 | Serpinb2 | 3.278788 |
| Cd36 | 1.128905 | Ak4 | 1.229102 | Flnc | 1.269286 |
| Odc1 | 1.842099 | Pcdh7 | 1.269592 | Wfdc17 | 1.456085 |
| Crybg2 | 2.465809 | Spp1 | 1.067197 | Gm23935 | 1.294284 |
| Gadd45b | 1.292871 | Ptgs2 | 2.894253 | Tmem170b | 1.093572 |
| Lpl | 1.583326 | Malt1 | 1.487226 | Malat1 | 1.212092 |
| Ccl9 | 1.453569 | Lpp | 1.127589 | Gm42793 | 1.13147 |
| Nos2 | 1.787487 | Zc3h12c | 1.191443 | Siglec1 | 1.076483 |
| Cpd | 1.320127 | Lars2 | 1.289687 |  | |
| Glrx | 1.224487 | Nfkbiz | 1.154748 |  |  |
| Dok2 | 1.001777 | Myo1d | 1.552151 |  |  |
| Acod1 | 1.969987 | Csf3 | 7.542571 |  |  |
| Tnf | 1.529886 | Fnbp1l | 1.004014 |  |  |
| Hspa4l | 1.167018 | Gfod1 | 1.276797 |  |  |
| Slc39a10 | 1.118619 | Socs3 | 1.666069 |  |  |
| Il1rl1 | 2.12708 | Spink5 | 2.378692 |  |  |
| Olfm1 | 1.026111 | Olfr933 | 1.620367 |  |  |
| Il1rn | 2.094613 | Calcrl | 1.295164 |  |  |

Supplement table 2.

RNA-seq analysis of the predicted expressed genes log 2 Fold changes. This analysis data was the *S. suieae* acetyl-xylogalactan 20 μg/ml treatment compared to the control in the gene fold change.

| Symbol | log2Fold  Change | Symbol | log2Fold  Change | Symbol | log2Fold  Change | Symbol | log2Fold  Change | Symbol | log2Fold  Change | Symbol | log2Fold  Change |
| --- | --- | --- | --- | --- | --- | --- | --- | --- | --- | --- | --- |
| Cd52 | 1.694742 | Nos2 | 2.752398 | Rgs16 | 3.938952 | Ldlr | 1.245878 | Txnip | 1.285585 | Clec7a | 1.108348 |
| Mmp14 | 3.092539 | Cpd | 1.735258 | Nek6 | 1.309475 | Myo1e | 1.125628 | Jarid2 | 1.423408 | D330050G23Rik | 2.414287 |
| Ccl3 | 2.440737 | Nr1d1 | 1.663052 | Lcn2 | 3.659359 | Ptgs2 | 4.676772 | Slco4a1 | 1.086931 | Tmem170b | 1.435641 |
| Tgfb1 | 1.065441 | Acly | 1.114508 | Olfm1 | 1.1513 | Nktr | 1.050536 | Fnbp1l | 1.35278 |  | |
| Cd36 | 1.610483 | Nfkbia | 1.293178 | Il1rn | 3.120886 | Cish | 6.418317 | Sh3pxd2b | 1.069513 |  |  |
| Etv3 | 1.435338 | Glrx | 1.735238 | Dhrs9 | 1.075287 | Malt1 | 2.814818 | Tet2 | 1.256581 |  |  |
| Ppfia3 | 2.390728 | Ngly1 | 1.824097 | Siglec1 | 1.499213 | Lss | 1.319424 | Kcnj2 | 1.119114 |  |  |
| Sqor | 1.014364 | Sh3bp5 | 1.050126 | Il1b | 8.57982 | Lpp | 1.463439 | Kdm7a | 1.08303 |  |  |
| Sod2 | 1.045414 | Dok2 | 1.115061 | Il1a | 5.573585 | Igf2bp2 | 1.431308 | Zc3h12a | 2.374287 |  |  |
| Bcl2l1 | 1.148013 | Acod1 | 3.279631 | Src | 1.597902 | Qsox1 | 1.033649 | Plaur | 2.371697 |  |  |
| Mrpl52 | 1.092345 | Dab2 | 1.099274 | Slc7a11 | 1.55591 | Dgkh | 1.614548 | Flrt2 | 1.131505 |  |  |
| Odc1 | 2.388719 | Gbe1 | 1.202698 | S100a11 | 1.133108 | Dhcr24 | 1.413911 | Socs3 | 3.231437 |  |  |
| Crybg2 | 2.936091 | Abcc1 | 1.0892 | Fubp1 | 1.064134 | Dcbld2 | 1.242135 | Spink5 | 3.029375 |  |  |
| Gadd45b | 2.579355 | Clec4n | 2.050468 | Tnc | 5.472446 | Zc3h12c | 1.467709 | Cxcl2 | 1.546699 |  |  |
| Lpl | 1.979827 | Prepl | 1.356494 | Ak4 | 1.505165 | Lars2 | 1.281053 | Layn | 1.686343 |  |  |
| Cd274 | 1.982101 | Mapk8ip3 | 1.055671 | Pdpn | 3.344452 | Nfkbiz | 1.387986 | Serpinb2 | 5.594589 |  |  |
| Il13ra1 | 1.075918 | Tnf | 2.088827 | Tnfrsf1b | 1.74018 | Ccl2 | 2.118863 | Dhrs3 | 1.552327 |  |  |
| Slc12a7 | 1.047889 | Cdk2ap2 | 1.589315 | Fgr | 1.219741 | Myo1d | 2.040564 | Slc31a2 | 1.408795 |  |  |
| Ccl6 | 3.563296 | Msr1 | 1.561837 | Cdk14 | 1.035436 | Btg1 | 1.104463 | Flnc | 1.654471 |  |  |
| Ccl4 | 1.464253 | Nrp2 | 1.14147 | Pcdh7 | 1.389642 | Micall2 | 1.568455 | Wfdc17 | 2.79966 |  |  |
| Ccl9 | 2.324454 | Slc39a10 | 1.123031 | Stap1 | 1.106808 | Csf3 | 10.8499 | Cebpd | 1.423551 |  |  |
| Srgn | 1.223481 | Cflar | 1.085641 | Spp1 | 1.327015 | F10 | 1.865481 | Gm23935 | 1.509648 |  |  |
| Plek | 1.431207 | Il1rl1 | 2.959355 | Furin | 1.448352 | Bcl2a1d | 2.00247 | Malat1 | 1.541133 |  |  |
| Rel | 1.238969 | Itgax | 3.249656 | Pim2 | 2.663138 | Gm29216 | 2.195671 | Sowahc | 1.658892 |  |  |

Supplement table 3.

RNA-seq analysis of the predicted expressed genes log 2 Fold changes. This analysis data was the *S. suieae* acetyl-xylogalactan 30 μg/ml treatment compared to the control in the gene fold change.

| Symbol | log2Fold  Change | Symbol | log2Fold  Change | Symbol | log2Fold  Change | Symbol | log2Fold  Change | Symbol | log2Fold  Change | Symbol | log2Fold  Change | Symbol | log2Fold  Change |
| --- | --- | --- | --- | --- | --- | --- | --- | --- | --- | --- | --- | --- | --- |
| Bcl6b | 2.475479 | Mmp9 | 1.015965 | Acod1 | 3.580979 | Siglec1 | 1.455418 | Itgax | 2.688765 | Myo1d | 2.415331 | Dhrs3 | 1.634531 |
| Itga5 | 1.026867 | Slc12a4 | 1.025184 | Dab2 | 1.30063 | Il1b | 9.685699 | Pim2 | 2.795799 | Btg1 | 1.006839 | Slc31a2 | 1.301445 |
| Cd52 | 1.8438 | Ccl6 | 3.171529 | Dcstamp | 1.344055 | Il1a | 6.753146 | Ldlr | 1.258445 | Micall2 | 1.675183 | Flnc | 1.567746 |
| Mmp14 | 2.695921 | Ccl4 | 1.943457 | Hcls1 | 1.264317 | Src | 1.611484 | Myo1e | 1.106573 | Rara | 1.403637 | Wfdc17 | 2.156307 |
| Ccl3 | 2.437671 | Ccl9 | 2.097489 | Abcc1 | 1.111123 | Slc7a11 | 1.572844 | Ptgs2 | 4.758139 | Csf3 | 11.85213 | Cebpd | 2.132156 |
| Acap1 | 1.284248 | Srgn | 1.239663 | Clec4n | 2.302808 | S100a11 | 1.268526 | Cish | 7.474571 | Jarid2 | 1.554126 | Cd80 | 1.450711 |
| Tgfb1 | 1.420415 | Plek | 1.318806 | Pim1 | 1.055792 | Lmna | 1.250399 | Fhl3 | 1.290647 | Slco4a1 | 1.366835 | Gm23935 | 1.250938 |
| Cd36 | 1.422032 | Phlda1 | 2.237713 | Tnf | 2.761263 | Bcar3 | 1.007987 | Malt1 | 2.442418 | Fnbp1l | 1.112112 | D330050G23Rik | 2.25379 |
| Etv3 | 1.131121 | Rel | 1.12885 | Pitpnm1 | 1.202382 | Nfkb1 | 1.134225 | Lss | 1.50341 | Rad54l2 | 1.086163 | Bcl2a1d | 1.851807 |
| Ier3 | 1.056924 | Upp1 | 3.573801 | Cdk2ap2 | 1.614856 | Tnc | 5.570737 | Rac2 | 1.02418 | Sh3pxd2b | 1.339256 | Cebpb | 1.193444 |
| Mvd | 1.148964 | Nos2 | 2.810889 | Msr1 | 1.320569 | Ak4 | 1.308146 | Igf2bp2 | 1.125714 | Stk40 | 1.103362 | Scimp | 3.62941 |
| Bcl2l1 | 1.356784 | Cpd | 1.704437 | Slc16a3 | 1.338449 | Pdpn | 3.122179 | Qsox1 | 1.00206 | Zc3h12a | 2.686963 | Cxcl2 | 1.980451 |
| Pou2f2 | 1.305678 | Acly | 1.154237 | Il1rl1 | 3.252696 | Tnfrsf1b | 1.772978 | Sik3 | 1.185593 | Hcar2 | 1.432168 | Calcrl | 1.966249 |
| Odc1 | 2.96834 | Nfkbia | 1.299971 | Rgs16 | 4.584738 | Fgr | 1.403883 | Pkd1l2 | 1.66802 | Plaur | 2.555413 | Layn | 1.334675 |
| Crybg2 | 3.316564 | Evl | 1.427279 | Nek6 | 1.009954 | Acot7 | 1.214401 | Dhcr24 | 1.757776 | Marcksl1 | 1.037585 |  | |
| Actn1 | 1.120661 | Tnfaip2 | 1.032673 | Lcn2 | 3.04816 | Pcdh7 | 1.503366 | Nfkbiz | 1.725935 | Eva1b | 4.011015 |  |  |
| Gadd45b | 1.975292 | Glrx | 1.974187 | Olfm1 | 1.342349 | Spp1 | 1.407527 | Ccl7 | 2.847589 | Gfod1 | 1.320429 |  |  |
| Lpl | 1.758878 | Ngly1 | 1.960255 | Traf1 | 1.409309 | Rhof | 1.501524 | Ccl2 | 3.030703 | Cd14 | 1.111241 |  |  |
| Ncf1 | 1.02473 | Sh3bp5 | 1.089702 | Il1rn | 3.294861 | Furin | 1.702414 | Tmem98 | 2.64528 | Socs3 | 3.883089 |  |  |
| Cd274 | 1.45519 | Dok2 | 1.653139 | Dhrs9 | 1.040273 | Rasgrp4 | 1.588214 | Serpinb2 | 5.545815 | Spink5 | 2.84686 |  |  |

Supplement 4. The HPLC of the polysaccharide composition.


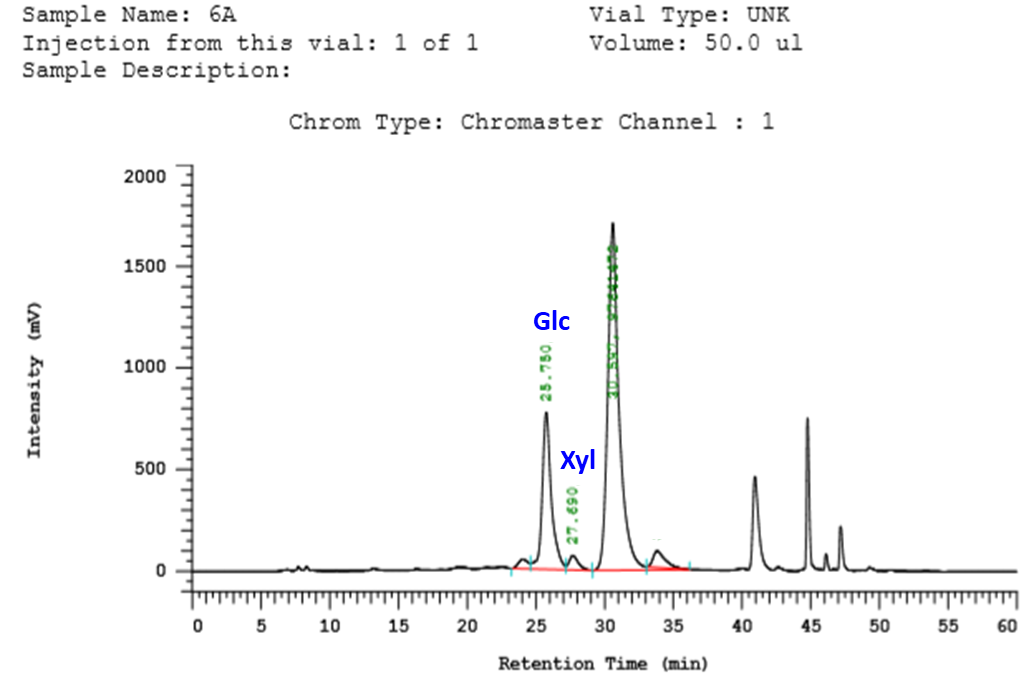


Supplement 5. The 2D NMR figure of the polysaccharide.


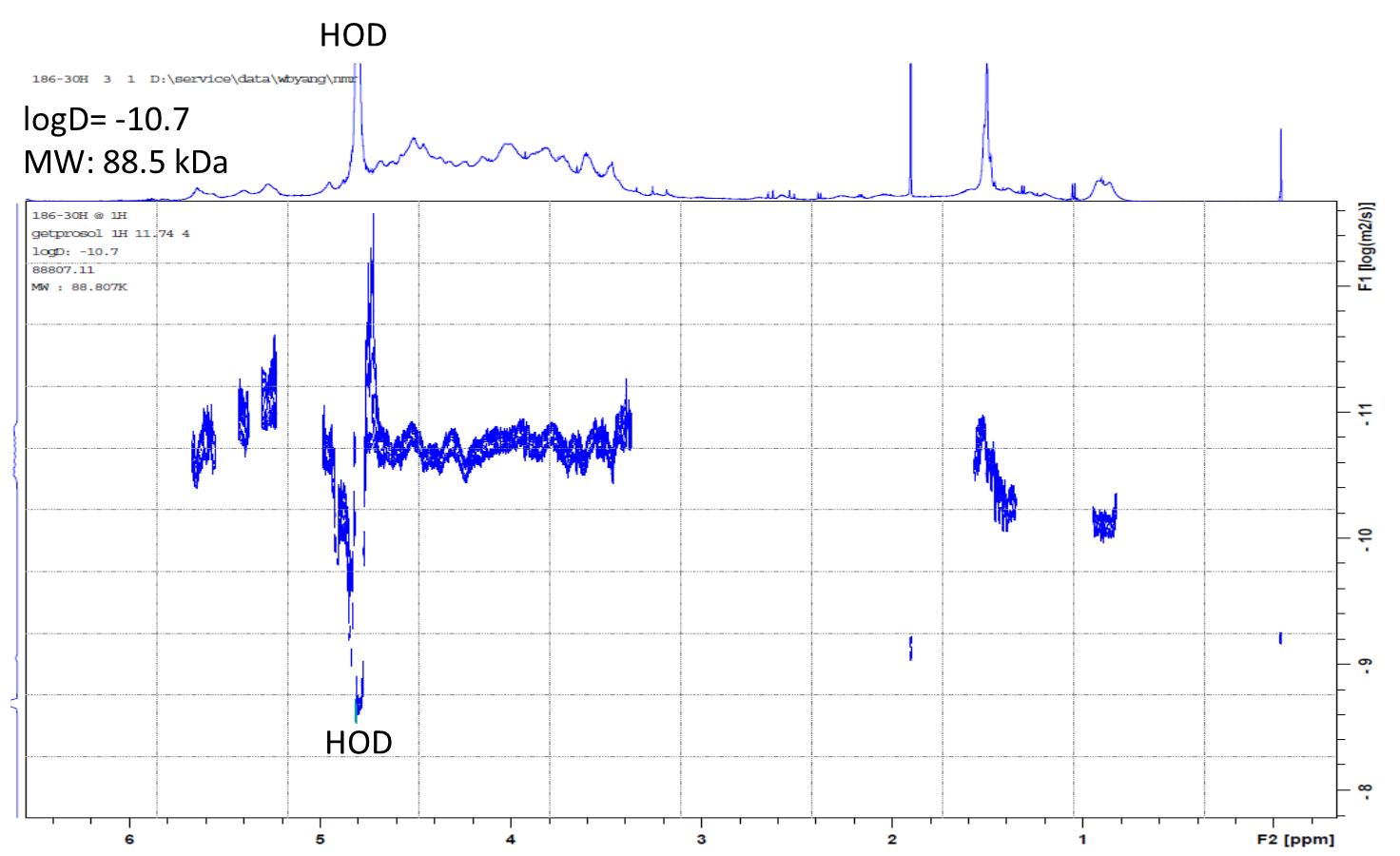


Supplement 6. The IR spectrum of acetyl xylogalactan.


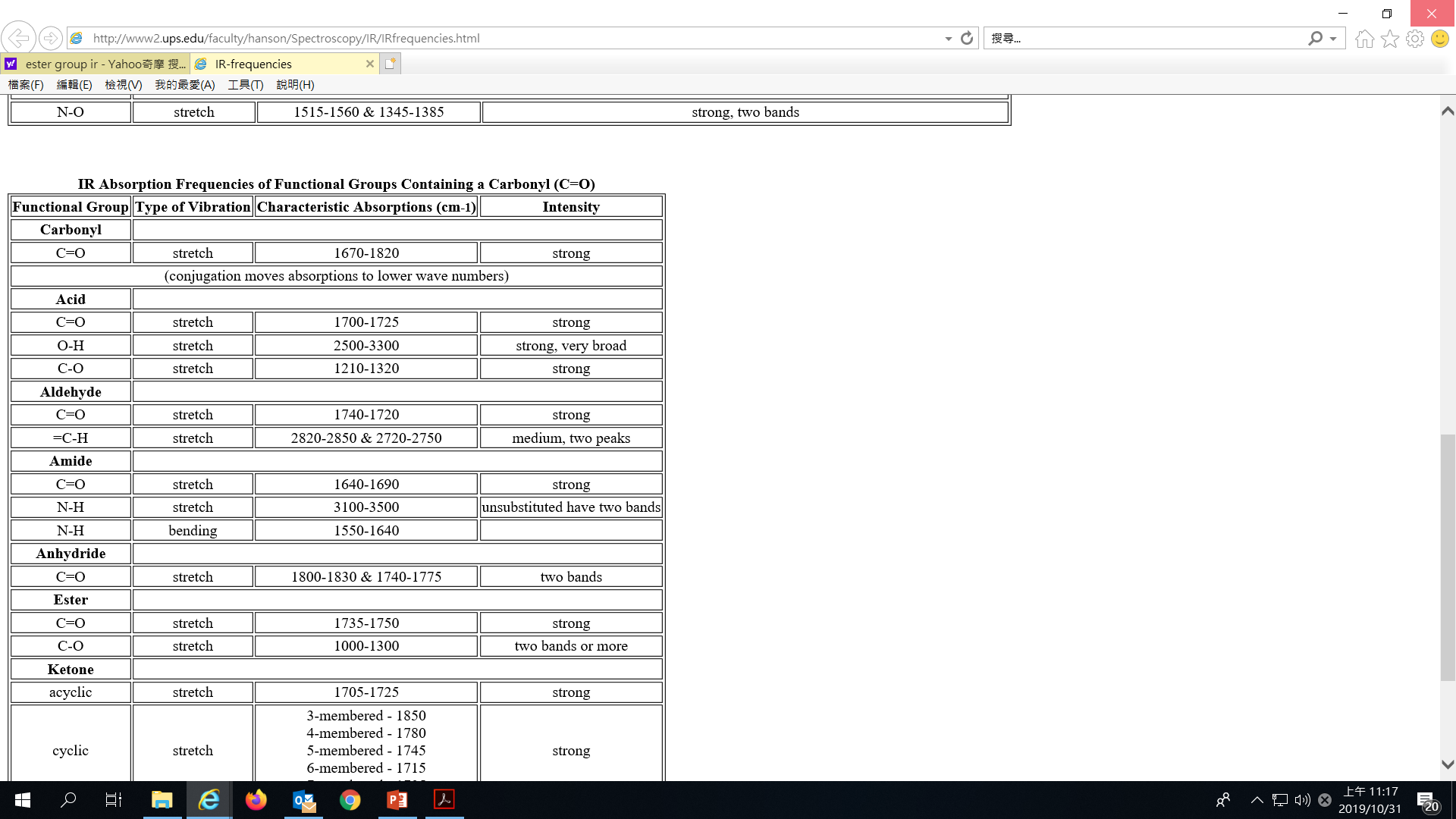


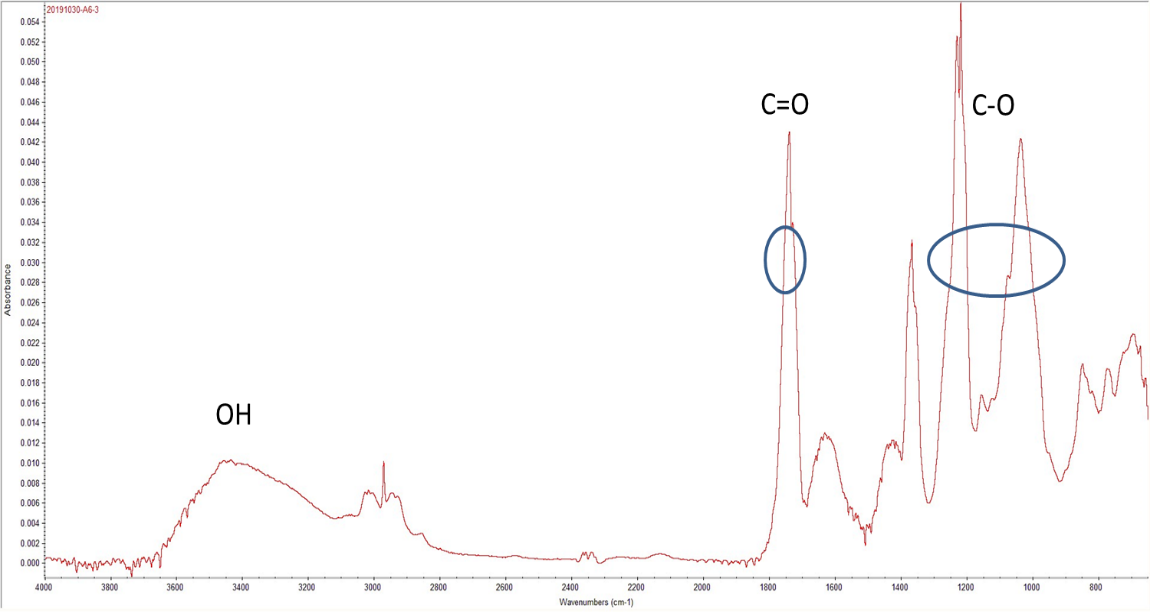


Supplement 7.

IL-6production from the RAW 264.7 macrophages pre-treated with or without *S. suieae* acetyl-xylogalactan following co-culture with LPS (100 μg/ml). 1 × 10^6^ RAW 264.7 macrophages were cultured in a 96-well plate with or without 10, 20, or 30 μg/mL *S. suieae* acetyl-xylogalactan for 24 h. Following to remove the culture medium, the 200 μl DMEM contained with LPS (100 μg/ml) was added to co-culture with the RAW264.7 for 24 h. The culture medium was then analysed using the ELISA IL-6 kit (QIAGEM, SEM03015A) at an OD of 450 nm. Scheffé’s test and one-way ANOVA were used to analyse the statistical significance between the treatment and control groups. A p-value of <0.05 was considered statistically significant as * marker. The result was presented with an enhancing effect of the RAW 264.7 pre-treated with polysaccharide to release the IL-6.


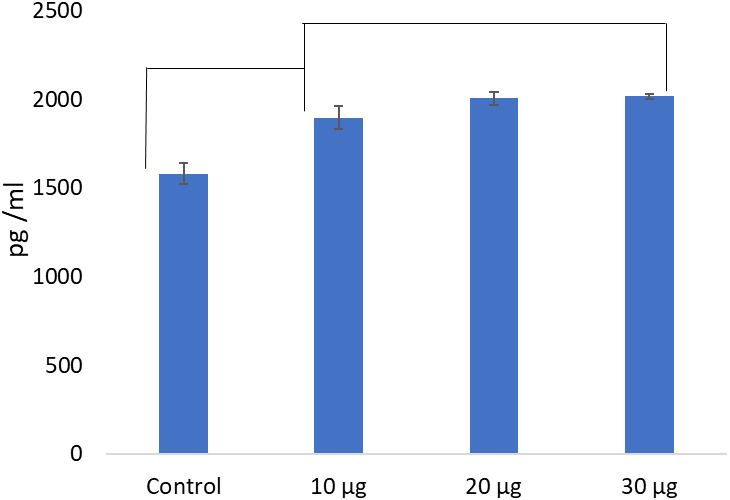


*
